# Prognosticating COVID Therapeutic Responses: Ambiguous Loss and Disenfranchised Grief

**DOI:** 10.3389/fpubh.2022.799593

**Published:** 2022-04-12

**Authors:** Harjinder Kaur-Aujla, Kate Lillie, Christopher Wagstaff

**Affiliations:** ^1^Institute of Clinical Sciences, University of Birmingham, Birmingham, United Kingdom; ^2^Institute of Clinical Sciences, Keele University, Keele, United Kingdom

**Keywords:** COVID, loss, grief, therapy, primary care, BAME

## Abstract

Conventionally, therapeutic assessments, interventions, and treatments have focussed on death-related “losses and grief” responses. It is purported that the COVID-19 aftermath has resulted in losses that cannot always be encapsulated using this method. In search of reasoning, models and theories that explain the sweeping mass destruction that COVID-19 has caused, key concepts arise in terms of how we should deal with losses and in turn support patients in the health and social care sector, (notwithstanding formal therapeutic services). There is a crucial need to embrace ambiguous loss and disenfranchised grief into everyday terminology and be acquainted with these issues, thereby adapting how services/clinicians now embrace loss and grief work. Integral to this process is to recognize that there has been a disproportionate impact on Black and minority ethnic communities, and we now need to ensure services are “seriously culturally competent.” Primary Care services/IAPT/health and social care/voluntary sector are all likely to be at the forefront of delivering these interventions and are already established gatekeepers. So, this article discusses the prognostic therapeutic response to non-death related losses and grief, not restricted to the formal echelons of therapeutic provision.

## Introduction: The Context

“...*in a field focused on the rupture in attachment bonds and associated grief in the aftermath of the death of a significant person, the far vaster domain of non-death losses has receded into relative invisibility, though the grief that attends them often may be equally substantial”. (1, p.12)*

There are many theories and subjugations about death, grief and loss, over the last Century. Harris (1) presents a comprehensive account of non-death related losses. This paper extends and adds to the valuable contributions due to the recent impact of COVID-19 and encourages health and social care professionals to be mindful of the insidious losses that may fester. Although this paper is written from a UK perspective the authors do not doubt that other cultures are undergoing similar traumatic challenges.

### Aim

The purpose of this article then is to highlight that grief work in therapeutic services has largely been on death, it is then hardly a revelation that we are now “pandemic stricken” to address the therapeutic aftermath of COVID-19. The primary aim is to deduce the literature on non-death related COVID and make recommendations to therapeutic providers to adapt traditional loss and grief interventions. This paper uniquely embraces the disproportionate impact on BAME (Black, Asian and Minority Ethnic) communities rather than an afterthought as seen in some articles, reminding scholars of the need to follow suit.

### Methods

A selective review of papers has been presented to illustrate the need for focus on non-death related losses due to the breadth of literature in many fields. This methodology demonstrates an alternative to systematic review which can often be constrained to specific subjects or fields and lend little to a newly developing topic, as such, COVID related non-death losses. It would be naïve to assume that currently, one central literature searching system can fully encapsulate such a new topic, particularly due to the multi-faceted nature of COVID impact from marketing to public health. The authors acknowledge that this process is not free from selective bias and indeed compromises the depth that a systematic review would capture. However, the idea is to encourage debate into the nature of COVID losses and advocate for further research.

## Results

This selective review enabled the deducing of a wide-range of factors that need to be accounted for when considering COVID related losses in therapeutic losses. The result would enable therapists and clinicians to ensure their assessments and treatment protocols moving forward look beyond death when deciphering COVID impact factors. Indeed, to ensure BAME consideration is not missed regarding access or adaptability of current workings.

## Discussion

The discussion embraces a wide plethora of multi-modal literature sources, reflecting on key areas that lend to the panacea of therapeutic advances notwithstanding the BAME interface and additional considerations. Explicit recommendations are made to the therapeutic community to adapt to non-death related losses, giving due consideration to BAME communities.

## A Public Health Issue

The perceived capricious and fickle nature of COVID and evolving evidence base has affected every corner of the globe with changing requirements to isolate; most pertinently it has left many families grieving loved ones and others dealing with the loss due to physical disability associated with long COVID (2). Almost everybody has experienced the loss of restricted movement and reduced social contact whilst others have faced redundancy or reduced income and boredom whilst on furlough. Many are also affected by contradictory news reports and national strategies leading to an insidious loss of trust. COVID-19 has indeed left people bereft of many less recognized losses that need to be acknowledged and given credibility. Moreover, therapeutic services need to embrace the “being present” and relational needs of the patient over the primary need for outcomes. There is now a pressing political, academic, and social need to look at non-related deaths, namely, ambiguous loss and disenfranchised grief.

COVID-19 is a human disaster that has led to a form of loss and grief that is complex and cannot fit traditional pathologies of depression, anxiety, and worry—the medical quandary of psychiatric diagnosis may fall short to address some of the idiosyncratic experiences of many. The resulting “new normal” of the COVID-19 pandemic has left families bewildered with traumatic losses as never before (3). At the time of writing 127,762,656 cases and 2,795,872 deaths from COVID-19 (4) have been reported throughout the world. These statistics are open to interpretation and should be used with caution due to the differences in reporting/ recording deaths, particularly in less developed countries such as India (5). The impact of COVID-19 on the world in terms of deaths alone is non-quantifiable.

COVID losses are a public health issue and the psychological processing of this cannot be clumped in with other models. However, it is widely acknowledged that mental health is a public health issue, and research has extensively identified that the impact of lockdown was severe (6, 7) and low mood identified as a real concern (8). The Afghanistani crisis that arose alongside the pandemic resulted in a secondary global public health concern and strategies to support these communities need to be considered (9).

The health emergency caused at the societal level has further marginalized ethnic minorities disproportionately, and the urban environment may critically influence the mental health of minorities during the pandemic (10). Pierce et al. (11) recommend that when considering the longitudinal impact of COVID-19 on mental health recommends prioritizing the needs of young people, women and pre-school children, and the authors wish to draw attention to the BAME aspect in light of this.

### Definitions

Defining loss and grief has always been fraught with semantics leading to various schools of thought about the differences in terminology, and to the extent to which it relates to death or other losses. Grief is frequently defined as the normal response to loss (12). Yet it is often inferred that death is not the only loss, but the only loss that legitimizes a grief reaction. According to Harris (1), a loss can be defined as an experience where there is a change in “circumstances, perception or experience,” and the individual is unable to return to a previous state. Her analogy of a shattered piece of glass that can be mended but never exists is useful in describing how loss is experienced. The idiographic positioning of the interpretation of loss in this definition is precisely what is needed to formulate the wide array of experiences that COVID-19 has inflicted on the population. Traditional approaches to grief have largely focussed on death and relational separation. The use of memory boxes and “digital death” literature posited important perspectives of keeping the “memories alive” or “celebrating the life and contributions,” they made to the idiographic journey. Digital methods of grief are particularly useful in helping young people grieve (13). The use of storytelling, reframing and creating a narrative of the deceased is considered a way of coping (14). The use of rituals around death have long been appraised and promoted to process death (15). Some of these interventions may have a place in formal therapeutic junctures and create some processing of unresolved losses related to COVID.

There have been multiple complex and nuanced understandings of grief. Grief has been conceptualized as a disease (16) and as as a series of stages in time (17, 18). Kubler Ross (19) famously formulated a five-stage theory of grieving based on her work talking to dying patients in a hospice setting. Although widely critiqued as presenting a simplistic and linear understanding of grief this model is widely used within therapy and has greatly influenced many practitioners' understanding. A preferred model is the Dual Process Model (20) which proposes that grief involves the individual in two separate but interrelated modes of grief. These are a “loss orientation” mode when the focus is on acknowledging and processing the emotions of grief and a “restoration orientation” mode when the focus is on engaging in coping with the world without the support of the deceased. Hence, the individual oscillates between periods of emotion and remembering and adjusting their lives. This model is preferred in terms of therapeutic psychoeducation or indeed managing COVID-19 related grief as it draws attention to the fact that isolation and lockdown have not only challenged individuals to process the emotions of grief without the physical presence of the extended family, friends, and professional support systems. This is epitomized by a new legal limit in the number of mourners at a funeral. However, it is also important to note that there have also been abundant challenges to restoration, requiring the bereaved to negotiate a world that is greatly altered without the support of those they loved.

It is acknowledged that not all griefs are equal or treated equally by society. Doka (21) defines disenfranchised grief as grief that results when a person experiences a significant loss and the resultant grief is not openly acknowledged, socially validated, or publicly mourned. In a time when many are grieving the unanticipated death of loved ones, others may feel inhibited and disenfranchised from expressing the grief they feel at not being able to visit living relatives, particularly those who are frail elderly and/or in residential care. Indeed, Sudnow (22), emphasized the concept of social death, in which the person is alive but treated as if dead. It is very easy to postulate that many individuals are feeling socially dead due to enforced restriction of social contacts currently. Another interrelated concept is that of ambiguous loss (23). This is a loss that occurs without closure or clear understanding. A commonly cited example is that of a spouse of a person with dementia, who is both with and simultaneously without, their partner. The loss of a relationship during COVID can similarly be conceptualized as ambiguous grief as the loved one is both living but unavailable to normal contact. Furthermore, it is important to acknowledge that there are liberating losses (24) when people experience feelings of relief, freedom, or happiness when society expects sadness and distress. This may relate to an acknowledgment of the end of suffering after the ambiguous loss of caring for someone with dementia but may also be experienced when COVID restrictions have prevented the necessity of visiting the sick and dying.

Much grief resolves or develops into an increased sense of maturity with time (18). However, a proportion of grief can be persistent and severely disabling. This is commonly termed complicated or complex grief. Factors that may be associated with complex grief include a sense of disbelief regarding the death, anger and bitterness regarding the death (25). There remain many COVID-19 deniers and the rapidness with which this new disease has impacted society and front-line workers mean that there is the potential for deaths and other losses associated with COVID-19 to linger without support and attention (26).

### Death and Loss

Ariès (27) highlights how death occurs in a historical, cultural, and social context. His seminal work, Western Attitudes Toward Death from the Middle Ages to the Present, presents four key periods in the past millennium. He documents an abrupt change in the mid 18th century when death becomes othered and dramatized. He highlights how family and friends have been enabled to mourn and freely display emotion from this time (27). Death separated the living from the dead and the living were consoled through memories and mourning. He then shows how death became more hidden and shameful in the 19th century and was increasingly associated with old age. Indeed, the majority of people began to die in hospital with shielding of information about the death to spare the bereaved from emotional turmoil. Jacobssen (28, 29) proposed a fifth, more recent phase of spectacular death, where death and mourning have increasingly become spectacles, with a strong urge to create memories and memorials, both physical and digital for those who have died. Walter (30) draws attention to the way that dying, funerals, mourning and afterlife beliefs are continually evolving and that we must evolve new ways to manage dying, funerals and mourning. Walter (30) highlights how containing grief and emotion can be psychologically and socially necessary in high death rate situations and societies whilst expressing them can be productive in times of peace and security. The challenges presented by COVID-19 as death again occurs sequestered in hospitals but mourning and grief are continually being discussed in the public sphere and the living are confronted with multiple ambiguous losses embedded in restrictions to everyday life within a multicultural society (31).

## The Medical Perspective-Diagnostics

We know that death alone is not the only event that can cause grief reactions, even complicated grief in people (32, 33). Studies are well averse to the aspect that ambiguous loss and disenfranchised grief in people can take a toll on their livelihoods, often undetected and minimized as “life- changes.” Prior attempts to capture difficulties in response to change have been encapsulated by “adjustment disorder” and focus on behaviors rather than the root cause. In psychiatry, the diagnosis of “Prolonged Grief disorder” (34) or “Persistent Complex Bereavement Disorder” (35), is fairly new. Prigerson et al. (36) developed a psychometric validity of criteria where there is a heightened risk of persistent “distress and function.” PTSD and the relationship with grief are considered yet, the psychological measures unless used in conjugation with grief measures do not necessarily look at loss. A history of losses can be mesmerized by depression and anxiety disorder, the patient not disclosing the losses and the therapist working on symptomatology. Nevertheless, Diagnostic Criteria for DSM-5 Persistent Complex Bereavement Disorder and ICD-11 Prolonged Grief Disorder criteria focus on the loss of a significant other by death. Harris (1) posits that in non-death losses the term chronic sorrow is more appropriate and points out key differences between the terminology. Inevitably, it means the presentations of COVID-19 losses in the form of chronic sorrow will need to be detected by front line workers and probably treated by primary care or voluntary sector talking therapy provision.

Essentially, chronic sorrow becomes an important consideration for how loss is assessed during and post-COVID, and how we capture the timeline in the therapeutic approaches whether they be counseling or CBT. The usual psychiatric evaluation or clinical presentation may need to be more investigative in nature, particularly where the presentation of the individual may be taciturn or morose. Not all individuals who experience grief and loss will likely be assessed or even formally diagnosed by a psychiatrist- many of these individuals will rely on a good formulation in psychological services and support from the wider health and social care services. Using a variety of psychological measures to triage, such as the Inventory of Complicated Grief (ICG) instead of usual trauma measures should be given due consideration (37, 38). All very well where there is grief, but more is needed in terms of designing psychological measures for the impact of COVID and less considered losses.

## COVID-19 Bottomless Pit of Losses

The challenges presented by COVID-19 as death again occurs sequestered in hospitals, but mourning and grief are continually being discussed in the public sphere and the living are confronted with multiple ambiguous losses embedded in restrictions to everyday life within a multicultural society (31).

Living in bubbles and self-isolating has affected previously formed collegial relationships and bonds. Anthologists have suggested the alternative approach of social containment, which is more about building links and reducing isolation (39, 40). The key areas of prospective lockdown striking have been identified, however notably, the losses are subject to individual interpretation and are endless. The profound impact on vulnerable and already disadvantaged groups in society cannot be quantified; the elderly, victims of abuse, disabled/ mental health affected, have all experienced the consequences of COVID-19 (41). The fragility of social interactions is further observed in the closure of shops, nightclubs, pubs, football stadiums and social venues in the UK, albeit arguably these closures could have been earlier.

Across the life- span, children have been adversely affected by the uncertainty that the Schools' debate has caused- whether children are at risk at school or not was always likely to be anxiety-provoking. The intersection with socio-economic status and that 50% of children did not get free school meals during the pandemic is extremely concerning (42). There will be a scientific evidence base to follow when children return to school and the impact of the pandemic. University numbers have been affected particularly where International students are concerned, and refunds are demanded where online provision is alternated at times (43). There is a whole population of students and pupils who will need support with educational loss in years to come, and services need to respond to these needs.

The UK employment market alone is radically affected by COVID-19 and despite support packages for SMEs (Small and Medium-size Enterprises), the business industry accounting for 50% of all UK revenue was badly affected by the pandemic (44). Notably, an approximate 5% proportion of this sector is likely to be occupied by BAME communities due to the discrimination they encounter in the labor market. Unemployment rates are high (5% compared to 4% pre-pandemic), benefit claimants have risen by 113% and the economic recession will inevitably affect the population substantially in due course.

Nicola et al. (45) provide a comprehensive global socio-economic analysis of the impact of COVID. The agricultural industry has taken a 20% hit due to hotels and restaurants closing. Demand for petrol has reduced and this is a major loss of income. Whilst furlough schemes and other incentives are in place to keep the economy going, the recession and impact of the economic losses are likely to affect the whole of the UK population for years ahead. The social drift theory including impact on mental health (46) suggests downward mobility will inevitably give rise to disenfranchised losses, including impact on mental health strain.

Child contact in normal cases has become strained with lockdown rules, impacting children who wish to spend time with both parents, and indeed with shielding grandparents (47). Wealthy citizens have been “banking on COVID” in divorce settlements to ensure they pay out less (48). Law firms have alluded to an increase in divorce cases however this is yet to be confirmed in the upcoming Census 2021 (49). What we do know is high street stores have jumped on the “COVID loss” industry and are selling divorce celebration products, so all is not lost, i.e., banners such as “newly unwed.” Regardless, for many, there may be a disenfranchised loss in terms of the relationship and celebrations may not be foremost and hence support services need to consider the variety of responses a loss of relationship could invoke aligned to grief theories already purported.

Many perspectives of death have been explored and how we cope with it culturally. There are various religious and cultural practices around the meaning of death. Mourning, the expression of sorrow for someone's death is an important cultural practice however limitations on the numbers at funerals, restrictions on indoor gatherings and COVID travel restrictions have led to funerals becoming “by invitation only” during the COVID pandemic have all interrupted the normal expressions of grief. Moreover, singing and chanting in public have also been banned. For many, including those of Southeast Asian heritage, the loss of shared cultural experiences of mourning has complicated grief responses. Asian communities rely on inter- familiar support and because of the high impact of BAME deaths that these communities particularly suffered. Notably, loneliness and isolation affected people of all cultures across the lifespan, from older adults to young children.

This disenfranchisement of mourning cultures may be amplified by the ambiguity of the loss in the context of the pandemic. Inevitably, the processing of losses during COVID-19 in other cultures is an important consideration. The political and social struggle against Xenophobia toward the Chinese population, and further demonstrated through the Black Lives Matters concerns, have highlighted the disproportionate “ambiguous” losses that BAME families face. Social distancing has caused immense losses in cultural celebrations and the notion of extended families in the South Asian future, thus far not represented in academic literature.

Virtual methods had implications for older BAME communities, often where English was a second language. This resulted in not only isolation that was strange in extended family structures but a lack of recognition during the pandemic and service provision to cope with the disenfranchised grief of not connecting with others.

## Disproportionate Impact

Essentially, Otu et al. (50) address the disproportionate impact death has had on “already vulnerable” BAME communties. The authors acknowledge that COVID-19 and BLM (Black Lives Matter) has given rise to the debate around whether the term BAME is suitable (51). The contention around this terminology is ongoing, however, it is important to note that academically it is useful at this stage to use the term in accessing literature on the topic. The current revival of “lay' and secularized practices of keeping loved one's memories alive including children are resurfacing in therapeutic responses to death. Every therapeutic community or service must develop rapid systems to embrace the aftermath of COVID related presentations and predict new ways of working. These in turn mean a review of how current responses to grief and loss may differ over the coming years, a bio-psycho-social consideration is much needed.

There have been long-lasting effects on all communities including mental health deterioration during lockdown (52). The BAME population in the UK stands at 14% based on the Census (53), yet Black African males are 4 times more likely to die due to COVID than their white counterparts reinforcing already recognized health and social disparities (49). Chaudhry et al. (54) postulate that the 64% BAME National Health Service (NHS) employed staff may have been deployed into high virus areas which resulted in disproportionate deaths. The ongoing impact of COVID-19 and the disproportionate impact on BAME families cannot be underestimated, “not only have Black, Asian and minority ethnic people been overexposed to contracting COVID-19, but the economic impact of the pandemic is also likely to disproportionately affect these communities too” [(55), p. 5]. Sze et al. (56) in a systematic review concluded that individuals of Black and Asian ethnicity are at increased risk of COVID-19 infection and Asians may be at higher risk of ITU admission and death. Specifically, the Racial Disparities Unit (RDU) found an alarming impact of COVID-19 on Bengali and Pakistani communities mainly due to multi-generational households (57). There is a disproportionate rate of deaths in these communities which must be considered and catered for, rather than the usual homogenisation of BAME communities.

## Resilience, Coping and Hope

There is a humanistic desire that a global tragedy like COVID-19 should result in enhanced community social cohesion and resilience (58). Empaths across the world, charity workers, keyworkers and religious aid all stepped up to buffer against losses. Despite the critique, in the UK there were pockets of financial support available to businesses, homeowners and employers. Yet, developing countries relied on spiritual strength and versatility to be resilient. The kind acts of individuals and organizations, and some infrastructure, have inspired hope and compassion. These altruistic human conditions serve as antidotes to the manifold losses experienced during the pandemic and must be acknowledged. Trzebiński et al. (59) posited that the key ingredients to being resilient in the pandemic were, (1) the influence of meaning in life, (2) life satisfaction, and (3) assumptions on world orderliness and positivity, all of which will result in lower stress and anxiety responses. How society will respond long term to the losses of COVID is yet to be determined. All models of grief acknowledge that grieving takes time. Klass et al. (60) highlight the need for continuing bonds with the deceased to support healthy grieving and resilience. It may be that society will need to develop civic forms of national memorialisation or that a more individualized approach will be more beneficial in the long term. However, the restoration orientation of Stroebe and Schuts model (20) highlights that the cost will not just be emotional but in enabling everyone in society with the skills to reorientate to a post COVID world.

The term thanatechnology (61) incorporates internet tools that can be utilized to commemorate the deceased in the form of a continuing bond and inadvertently become digitally immortal. Testoni et al. (62) carried out a qualitative study in Italy during the pandemic to report the use of social networks to connect with others during the pandemic was useful in the mourning process. Features of ambiguous loss were present amongst those unable to be present with family, yet the connectedness was helpful. The feeling of abandonment by health services was a prominent theme within the study.

Furthermore, emotional dysregulation (the ability to regulate emotion to situations and adapt), has been considered to affect people across the life- span. Moccia et al. (63), in an Italian study, were able to identify the relationship between lack of emotional regulation and reduced hedonic tone (ability to feel pleasure) as predictors for COVID- related depression. Janiri et al. (64, 65) identified that emotional regulation was particularly a concern in older adults, over the age of 60 which resulted in low mood. Janiri et al. (64, 65) identified that emotional dysregulation was a key mediating factor when considering trauma in children. In fact, childhood trauma was seen to increase vulnerability to stress due to COVID-19. Therefore, it seems that emotional dysregulation and reduced hedonic tone should be targeted in therapeutic interventions especially for both older and younger age individuals to build resilience.

## Impact on Nurses

Healthcare professionals, including nurses, had to support increased numbers of people dying in all locations: Indeed, there were 76,000 excess deaths in England and Wales in 2020 (66). Home deaths increased by a third during COVID meaning community nurses were providing complex care to people who might otherwise have died in hospital. Deaths in hospitals also increased (66) with restricted family access, which has compounded the grieving experience.

Wilson and Kirshbaum (67) noted that over half of the deaths in the UK occur in NHS hospitals. Furthermore, bearing witness to these deaths and with the extra COVID related deaths were bound to have a profound impact on nurses. Al Thobaity and Alshammari (68) explored the emotional pressures that nurses underwent through the pandemic, often under-resourced and carrying out their roles without PPE (Personal Protective Equipment). According to Purba (69), nurses played an important role in managing COVID-19 on the frontline and should be supported to manage health inequalities.

The concept of vicarious trauma (secondary trauma) has been explored in nursing for a long time and the key impact from deaths and abuse-related risks accorded even in a research capacity, Taylor et al. (70). Sabo (71) argues that non-resolved compassion fatigue/burnout/stress results in vicarious trauma, and this is a concern. There is a pressing need then for nurses to develop self-care strategies to cope with the aftermath of the pandemic, namely, moral distress and injury (72, 73). Nurses are likely to require explicit support around loss and grief in response to the pandemic and services need to embrace the prospect and design services accordingly.

## Professional Considerations

Services need to develop robust Employment Assistance Programmes to ensure there are therapeutic services in place to support frontline staff and key workers both with the personal and professional impact of COVID. The concept of vicarious trauma is fraught with complications—an area that comes up commonly in therapy and professionals need to fully support staff to access therapy to improve patient care and reduce transference of loss between homework. The use of clinical supervision in medical professions needs to be utilized more effectively than ever, and supervisors need to have sufficient training not just in traditional grief but the entangled weave of ambiguous loss and disenfranchised grief (74).

Improving Access to Psychological Therapies (IAPT) services provide primary care psychological services within mental health in the UK and would be in a prime position to create impetus in what is considered as in “vogue” treatments for loss and grief. Currently, the UK offers services to children with mental health issues in secondary care only, and the demand is excessive. IAPT talking therapy services are likely to extend to children soon, perfect timing to catch the children affected by the nuances of COVID-19.

Grief theories have suggested that perhaps an old wound is reopened, and loss (ambiguous/disenfranchised or otherwise) is re-triggered. However, when multiple support mechanisms are available that compound the loss such as socialization, spirituality and community/family support this is then less prominent. The oscillation between good and bad days do eventually subside to become less distressing and frequent. How does and will this concept help to formulate and treat a post-COVID generation who have suffered months of ambiguous loss and perhaps actual loss?

Frontline service and need to develop loss and grief based Cognitive Behavioral Therapy (CBT) interventions for patients including flexible access to therapy for key-workers and shift workers outside of the usual office hours. The first author developed a well appraised virtual twilight service during the pandemic for a UK based service, and the uptake from essential workers was vast.

Moving away from the Kubler and Ross model and capturing ambiguous loss and disenfranchised grief using trauma and grief measures much like for any human catastrophe, may be the answer. The initial minimum data set required in these services to measure depression and anxiety and trauma is measured through the Impact of Events scale (IES). The Inventory of Complicated Grief (ICG) is not a part of IAPT indicators, and there is now a pressing need for this to be included (36–38). Furthermore, the identification of traumatic loss as part of a PTSD formulation is the best way to encapsulate the current paradigm and apply it to existing resources. Therapists at primary and secondary care levels must be trained to recognize and treat ambivalent loss and disenfranchised grief at this time of adversity. The target-driven services may have to adopt this method of delivery where loss is concerned. Harris posits that training programs that focus on “curing” and “fixing” individuals are insufficient. The need for a validating and compassionate response where the clinician is present with the client during the process rather than outcome-driven is emphasized.

The long-standing application of adapting current interventions have been applied to loss where there is not a physical loss and there may be workforce training needs to consider in time for the post lockdown influx of help-seeking individuals.

## Therapeutic Approaches

IAPT within primary care services use psychological models to train therapists using a scientific-practitioner model referred to as the Boulder model. Nathan (75) states CBT clinicians rarely use research to further the scientific-practitioner model and empirically supported treatments are encouraged. Professionally we need to plan and ensure workplaces and training educational establishments are training their workforce to deal with ambiguous grief and disenfranchised loss. Not having access to usual activities affects people in many ways, and we have a generation of post-COVID survivors who will need support to overcome this. Embedded within the infrastructure, the needs of BAME communities who are disproportionately affected must be foremost in any service delivery model. Harris (1) makes important recommendations on supporting a person through loss and “shattered dreams” and avoiding the “get over it and move on” Western approach to non-death losses. This is likely to be a lifelong adjustment to loss, and the target-driven services may have to adopt this method of delivery where loss is concerned. Harris posits that training programs that focus on “curing” and “fixing” individuals are ineffective. The need for a validating and compassionate response where the clinician is present with the client is emphasized.

A transdiagnostic and relationally driven model of assisting post-COVID generation needs to be developed for those who experienced loss is key to developing an understanding, and treatment packages for this population. Humphrey and Zimpfer (12), using a counseling approach, have presented useful methods to assess grief using the bio-psycho-social model, including spiritual and philosophical reasoning, and duly considers past “losses” to gain an understanding of current interpretation using a timeline approach. Losses across the lifespan may be key in understanding idiosyncratic responses to COVID−19, and this method of formulation presents intrigue and a focus on what is unanswered for the individual. The work of Humphrey and Zimpfer (12) is appraised to include the impact of anticipatory grief, and traumatic grief because of murder for example.

CBT does situate well with grief and loss, in theory, the use of cognitive meaning is at the forefront (76). In dealing with complicated grief, the facets of irrational cognitions, autobiographical memory, and coping strategies are seen as predictors (77, 78). The work of Mueller (79) in CBT traumatic grief is noteworthy. She recognizes the impact of COVID and embraces this into CBT. Rosner et al. (80) consider the application of CBT to complicated grief and this again can be adapted to COVID-19 losses. Working with the patient to stabilize, explore, and confront the most painful aspects of the loss are encouraged. Comprehensively, they posit a cognitive behavioral treatment manual for complicated grief disorder (CG-CBT) of 25 individual sessions. The longevity of treatment may be a concern in the implementation where shorter-term CBT or counseling therapy is considered in primary care services at least. Shear et al. (25) proposed a 16-session model of Complicated Grief Therapy (CGT), which may be a preferred protocol and proved more effective than interpersonal therapy in the RCT.

The debate between CBT or counseling for grief and/or loss has long eroded-both have a role and are equipped to deal with the aftermath of COVID. The evidence base for the therapeutic approach for COVID related losses is out there; it is now a case of service providers explicitly adapting and incorporating it into practice.

## Conclusion

There is a wealth of evidence that COVID-19 has resulted in unimaginable losses to the indigenous population, but even more so to the BAME community. It is anticipated that having considered the COVID related losses that may be less obvious, that of ambiguous loss and disenfranchised grief, professionals and services can respond to this efficiently. The need to be culturally competent due to the disproportionate impact on BAME communities is pressing. Planning mainstream services and responses to COVID losses for BAME communities need to be embedded in service development and not an afterthought (see Table 1). We now need prognostic therapeutic approaches to ensure loss and grief interventions are not limited to death alone and be adaptive to the COVID generation. Essentially, the therapeutic costs of COVID-19 “loss and grief” are idiosyncratic, and therefore the treatment of it remains at the intersection of therapeutic understanding by service providers and employers.

**Table 1:** Recommendations—responding to COVID losses.

**Recommendations**
• Embracing COVID-related ambiguous losses and disenfranchised grief into therapeutic provision including health and social care support regimes, which are less outcome orientated and focus on relational support.
• Incorporate the dual processing model into grief and loss training programmes on COVID-19 aftermaths, not exclusive to death.
• Consider relational trauma-informed grief interventions (including measures) more readily when assessing the impact of COVID-related losses, particularly within primary care IAPT services.
• Ensure robust key worker Employment Assistance Programmes to support the infrastructure and keyworkers affected by COVID-19.
• Emotional dysregulation and reduced hedonic tone should be targeted in therapeutic interventions across the lifespan.
• Proactively engage with BAME communities who may be less inclined to access therapies post-COVID, ensuring bi-lingual provision and acknowledge the enriched cultural affinity with spirituality and religion.
• Additional support for services to engage with BAME communities and recruit. Train representative clinicians that understand the culture and/or training in cultural competency.
• Capacity build within mainstream therapeutic services to address their needs, instead of creating fragmented/ ghettoization of new services or specialized “ethnic services,” which can create divisions.

## Author Contributions

All authors listed have made a substantial, direct, and intellectual contribution to the work and approved it for publication.

## Conflict of Interest

The authors declare that the research was conducted in the absence of any commercial or financial relationships that could be construed as a potential conflict of interest.

## Publisher's Note

All claims expressed in this article are solely those of the authors and do not necessarily represent those of their affiliated organizations, or those of the publisher, the editors and the reviewers. Any product that may be evaluated in this article, or claim that may be made by its manufacturer, is not guaranteed or endorsed by the publisher.

## References

[1] Harris DL (Ed.). Non-Death Loss and Grief: Context and Clinical Implications. 1st ed. London: Routledge (2019).

[2] Suleman M Sonthalia S Webb C Tinson A Kane M Bunbury S. Unequal Pandemic, Fairer Recovery: The COVID-19 Impact Inquiry Report. London: Health Foundation (2021).

[3] Walsh F. Loss and resilience in the time of COVID-19: meaning making, hope, and transcendence. Fam Process. (2020) 59:898–911. 10.1111/famp.12588 32678915 PMC7405214

[4] Worldometers. Coronavirus Update (Live) 2021 127,762,656 Cases and 2,795,872 Deaths from COVID-19 Virus Pandemic - Worldometer (2021).

[5] Bharati S Batra R. How misuse of statistics can spread misinformation: a study of misrepresentation of COVID-19 data. Available online at: rahulbatra.in/assets/paper.pdf (2021).

[6] Fiorillo A Sampogna G Giallonardo V Del Vecchio V Luciano M Albert U. Effects of the lockdown on the mental health of the general population during the COVID-19 pandemic in Italy: results from the COMET collaborative network. Eur Psychiatry (Italy), (2020) 63:e87. 10.1192/j.eurpsy.2020.89 32981568 PMC7556907

[7] Kaur-Aujla H Lillie K Wagstaff C. Oral paper; Prognosticating Covid-19 therapeutic responses: a bottomless pit of ambiguous loss and disenfranchised grief. In: Eur Conference on Mental Health (ECMH) (2021).

[8] Moura AAM Bassoli IR Silveira BVD Diehl A Santos MAD Santos RAD. Is social isolation during the COVID-19 pandemic a risk factor for depression? Rev Bras Enferm. (2022) 75(Suppl 1):e20210594. 10.1590/0034-7167-2021-0594 35262601

[9] Kaur-Aujla H Wagstaff C. Embracing Afghanistan's Refugees into the UK's Health and Social Care System. London: BMJ Publishing Group Ltd. (2021).

[10] Menculini G Bernardini F Attademo L Balducci PM Sciarma T Moretti P. The influence of the urban environment on mental health during the COVID-19 pandemic: focus on air pollution and migration-a narrative review. Int J Environ Res Public Health. (2021) 18:3920. 10.3390/ijerph18083920 33917942 PMC8068323

[11] Pierce M Hope H Ford T Hatch S Hotopf M John A. Mental health before and during the COVID-19 pandemic: a longitudinal probability sample survey of the UK population. Lancet Psychiatry. (2020) 7:883–92. 10.1016/S2215-0366(20)30308-4 32707037 PMC7373389

[12] Humphrey GM Zimpfer DG. Counselling for Grief and Bereavement. London: SAGE (1996).

[13] Abidin C. Young People and Digital Grief Etiquette. A networked Self and Birth, Life, Death. London: Routledge (2018). p. 160–74.

[14] Walter T. A new model of grief: bereavement and biography. Mortality. (1996) 1:7–25.

[15] Lindemann E. Symptomatology and recovery from acute grief. Am J Psychiatry. (1944) 101:141–8. 10.1176/ajp.101.2.141 8192191

[16] Engel GL. Is grief a disease? A challenge for medical research. Psychosom Med. (1961) 23:18–22. 10.1097/00006842-196101000-00002 13696798

[17] Bowlby J. Attachment and loss. New York, NY: Basic Book Company (1969).

[18] Oyebode J. On bereavement: studies of grief in adult life by Colin Murray Parkes. Br J Psychiatry. (2014) 205:213. 10.1192/bjp.bp.113.14313116477190

[19] Kubler Ross E. On Death and Dying. London: Routledge (1973).

[20] Stroebe M Schut H. The dual process model of coping with bereavement: rationale and description. Death Stud. (1999) 23:197–224. 10.1080/074811899201046 10848151

[21] Doka K. Disenfranchised grief in historical and cultural perspective. In: Stroebe MS Hansson RO Schut HE Stroebe WE editors. Handbook of Bereavement Research and Practice: Advances in Theory and Intervention. Washington, DC: American Psychological Association (2008). 10.1037/14498-011

[22] Sudnow D. Passing on, the Social Organization of Dying. Englewood Cliffs: Prentice Hall (1967).

[23] Boss P. Ambiguous Loss: Learning to Live With Unresolved Grief. Cambridge, MA: Harvard University Press (2009).

[24] Elison J McGonigle C. Liberating Losses: When Death Brings Relief. Cambridge, MA: Perseus (2003).

[25] Shear K Frank E Houck PR Reynolds CF III. Treatment of complicated grief: a randomized controlled trial. JAMA. (2005) 293:2601–8. 10.1001/jama.293.21.2601 15928281 PMC5953417

[26] Hinsliff G. Doctors Are Our Frontline Against COVID. Now They Lead the Fight Against Its Deniers, too. The Guardian Life Insurance Company of America. London: Guardian News & Media Limited (2021).

[27] Ariès P. Western Attitudes Toward Death: From the Middle Ages to the Present. Baltimore, MD: JHU Press (1975).

[28] Jacobsen MH. Spectacular Death—proposing a new fifth phase to Philippe Ariès's admirable history of death. Humanities. (2016) 5:19. 10.3390/h5020019

[29] Jacobsen MH. The Age of Spectacular Death. London: Routledge (2020).

[30] Walter T. The Revival of Death: Two Decades on 2014. Available online at: http://endoflifestudies.academicblogs.co.uk/the-revival-of-death-two-decades-on-by-tony-walter/ (accessed February 20, 2021).

[31] Jacobsen MH Petersen A. The return of death in times of uncertainty—A sketchy diagnosis of death in the contemporary “corona crisis.” *Soc Sci*. (2020) 9:131. 10.3390/socsci9080131

[32] Doka KJ. Silent sorrow: grief and the loss of significant others. Death Stud. (1987) 11:455–69. 10.1080/07481188708252210

[33] Doka KJ. Disenfranchised Grief: New Directions, Challenges, and Strategies for Practice. Illnois, IL: Research PressPub (2002).

[34] World Health Organization. International Classification of Diseases for Mortality and Morbidity Statistics (11th Revision). Geneva: World Health Organization (2018).

[35] American Psychiatric Association (APA). DSM-V Diagnostic and Statistical Manual of Mental Disorders. 5th ed. Washington, DC: American Psychiatric Association (2013). 10.1176/appi.books.9780890425596

[36] Prigerson HG Maciejewski PK Reynolds CF Bierhals AJ Newsom JT Fasiczka J. Inventory of complicated grief: a scale to measure maladaptive symptoms of loss. Psychiatry Res. (1995) 59:65–79. 10.1016/0165-1781(95)02757-2 8771222

[37] Prigerson HG Bierhals AJ Kasl SV Reynolds CF III Shear MK Newsom JT. Complicated grief as a disorder distinct from bereavement-related depression and anxiety: a replication study. Am J Psychiatry. (1996) 153:1484–6. 10.1176/ajp.153.11.1484 8890686

[38] Prigerson HG Horowitz MJ Jacobs SC Parkes CM Aslan M Goodkin K. Prolonged grief disorder: psychometric validation of criteria proposed for DSM-V and ICD-11. PLoS Med. (2009) 6:e1000121. 10.1371/journal.pmed.1000121 19652695 PMC2711304

[39] Barton C. Lockdown, loss and adaptation. J Aesthet Nurs. (2020) 9:227. 10.12968/joan.2020.9.6.227

[40] Long N. From social distancing to social containment. Med Anthropol Theor. (2020) 7:247–60. 10.17157/mat.7.2.791

[41] Economic and Research Council. Impacts of Social Isolation Among Disadvantaged and Vulnerable Groups During Public Health Crises. London: Economic and Social Research Council (2020).

[42] Parnham JC Laverty AA Majeed A Vamos EP. Half of children entitled to free school meals did not have access to the scheme during COVID-19 lockdown in the UK. Public Health. (2020) 187:161–4. 10.1016/j.puhe.2020.08.019 32980783 PMC7447260

[43] Dennis MJ. COVID-19 will accelerate the decline in international student enrollment. Recruiting Retaining Adult Learn. (2020) 22:1–7. 10.1002/nsr.30639

[44] McKinsey. Survey 2020 How the COVID-19 Crisis Is Affecting UK Small and Medium Size Enterprises. Available online at: mckinsey.com (accessed March 20, 2021).

[45] Nicola M Alsafi Z Sohrabi C Kerwan A Al-Jabir A Iosifidis C. The socio-economic implications of the coronavirus pandemic (COVID-19): a review. Int J Surg. (2020) 78:185–93. 10.1016/j.ijsu.2020.04.018 32305533 PMC7162753

[46] Fox JW. Social class, mental illness, and social mobility: the social selection-drift hypothesis for serious mental illness. J Health Soc Behav. (1990) 31:344–53. 10.2307/2136818 2135936

[47] Dalton L Rapa E Stein A. Protecting the psychological health of children through effective communication about COVID-19. Lancet Child Adolesc Health. (2020) 4:346–7. 10.1016/S2352-4642(20)30097-3 32243784 PMC7270522

[48] Financial times. Wealthy Use Covid Uncertainty to Challenge Divorce Settlements. London: Financial Times (2020).

[49] Public Health England. Disparities in the Risk and Outcomes of COVID-19. London: PHE Publications (2020).

[50] Otu A Ahinkorah BO Ameyaw EK Seidu AA Yaya S. One country, two crises: what Covid-19 reveals about health inequalities among BAME communities in the United Kingdom and the sustainability of its health system? Int J Equity Health. (2020) 19:189. 10.1186/s12939-020-01307-z 33109197 PMC7590239

[51] Khunti K Routen A Pareek M Treweek S Platt L. The language of ethnicity. BMJ. (2020) 371:m4493. 10.1136/bmj.m4493 33229395

[52] Daly M Sutin AR Robinson E. Longitudinal changes in mental health and the COVID-19 pandemic: evidence from the UK Household Longitudinal Study. Psychol Med. (2020) 13:1. 10.1017/S0033291720004432 33183370 PMC7737138

[53] Office of National Statistics (ONS). Census Analysis. London: Office for National Statistics (2011).

[54] Chaudhry FB Raza S Raja KZ Ahmad U. COVID 19 and BAME health care staff: wrong place at the wrong time. J Glob Health. (2020) 10:020358 10.7189/jogh.10.020358 33110554 PMC7563090

[55] Lawrence D. An Avoidable Crisis. (2021). Available online at: nhsbmenetwork.org.uk (accessed February 20, 2021).

[56] Sze S Pan D Nevill CR Gray LJ Martin CA Nazareth J. Ethnicity and clinical outcomes in COVID-19: a systematic review and meta-analysis. Eclinicalmedicine. (2020) 29:100630. 10.1016/j.eclinm.2020.100630 33200120 PMC7658622

[57] HM Government. Race Disparity Unit: Second Quarterly Report on Progress to Address COVID-19 Health Inequalities. (2021). Available online at: http://www.gov.uk/government/organisations/race-disparity-unit (accessed July 22, 2021).

[58] Townshend I Awosoga O Kulig J Fan H. Social cohesion and resilience across communities that have experienced a disaster. Nat Hazards. (2015) 76:913–38. 10.1007/s11069-014-1526-4

[59] Trzebiński J Cabański M Czarnecka JZ. Reaction to the COVID-19 pandemic: the influence of meaning in life, life satisfaction, and assumptions on world orderliness and positivity. J Loss Trauma. (2020) 25:544–57. 10.1080/15325024.2020.1765098

[60] Klass D Silverman PR Nickman S. Continuing Bonds: New Understandings of Grief. London: Taylor & Francis (2014). 10.4324/9781315800790

[61] Sofka CJ. Social support “internetworks,” caskets for sale, and more: thanatology and the information superhighway. Death Stud. (1997) 21:553–74. 10.1080/074811897201778 10179827

[62] Testoni I Azzola C Tribbia N Biancalani G Iacona E Orkibi H. The COVID-19 disappeared: from traumatic to ambiguous loss and the role of the Internet for the bereaved in Italy. Front Psychiatry. (2021) 12:620583. 10.3389/fpsyt.2021.620583 34025466 PMC8138554

[63] Moccia L Janiri D Giuseppin G Agrifoglio B Monti L Mazza M. Reduced hedonic tone and emotion dysregulation predict depressive symptoms severity during the COVID-19 outbreak: an observational study on the Italian general population. Int J Environ Res Public Health. (2020) 18:255. 10.3390/ijerph18010255 33396363 PMC7795888

[64] Janiri D Kotzalidis GD Giuseppin G Molinaro M Modica M Montanari S. Psychological distress after Covid-19 recovery: reciprocal effects with temperament and emotional dysregulation. An exploratory study of patients over 60 years of age assessed in a post-acute care service. Front Psychiatry. (2020) 60:590135. 10.3389/fpsyt.2020.590135 33304286 PMC7693440

[65] Janiri D Moccia L Dattoli L Pepe M Molinaro M De Martin V. Emotional dysregulation mediates the impact of childhood trauma on psychological distress: first Italian data during the early phase of COVID-19 outbreak. Aust N Z J Psychiatry. (2021) 55:1071–8. 10.1177/0004867421998802 33715469

[66] Office of National Statistics (ONS) (2021). Available online at: www.ons.gov.uk/peoplepopulationandcommunity/ (accessed July 26 2021).

[67] Wilson J Kirshbaum M. Effects of patient death on nursing staff: a literature review. Br J Nurs. (2011) 20:559–63. 10.12968/bjon.2011.20.9.559 21647017

[68] Al Thobaity A Alshammari F. Nurses on the frontline against the COVID-19 pandemic: an integrative review. Dubai Med J. (2020) 3:87–92. 10.1159/00050936134182609

[69] Purba AK. How should the role of the nurse change in response to Covid-19? Nurs Times. (London) (2020) 116:25–8.33387382

[70] Taylor J Bradbury-Jones C Breckenridge JP Jones C Herber OR. Risk of vicarious trauma in nursing research: a focused mapping review and synthesis. J Clin Nurs. (2016) 25:2768–77. 10.1111/jocn.13235 27161657

[71] Sabo B. Reflecting on the concept of compassion fatigue. J Issues Nurs. (2011) 16:1. 10.3912/OJIN.Vol16No01Man01 21800932

[72] Aafjes-van Doorn K Békés V Prout TA Hoffman L. Psychotherapists' vicarious traumatization during the COVID-19 pandemic. Psychol Trauma. (2020) 12(S1):S148–50. 10.1037/tra0000868 32478559

[73] Hossain F Clatty A. Self-care strategies in response to nurses' moral injury during COVID-19 pandemic. Nurs Ethics. (2021) 28:23–32. 10.1177/0969733020961825 33124492 PMC7604672

[74] Driscoll J Stacey G Harrison-Dening K Boyd C Shaw T. Enhancing the quality of clinical supervision in nursing practice. Nurs Stand. (2019) 34:43–50. 10.7748/ns.2019.e11228 31468814

[75] Nathan PE. The Boulder model: a dream deferred—or lost? Am Psychol. (2000) 55:250–2. 10.1037/0003-066X.55.2.250 10717975

[76] Matthews LT Marwit SJ. Complicated grief and the trend toward cognitive-behavioral therapy. Death Stud. (2004) 28:849–63. 10.1080/07481180490490924 15493080

[77] Nagy D Szamosközi S. The relationship between irrational cognitions, autobiographical memory, coping strategies and complicated grief. Procedia Soc Behav Sci. (2014) 127:524–8. 10.1016/j.sbspro.2014.03.303

[78] Nagy D Szamosközi S. Open Access Government 2021 UN Government Report Finds “Alarming” COVID Impact in South Asians. (2014). Available online at: https://www.openaccessgovernment.org/covid-impact-in-south-asians/104804/# (accessed February 20, 2021).

[79] Mueller M. Adapting CBI. (2021). Available online at: https://www.octc.co.uk/workshops/adapting-cbi-for-people-with-asd-2-2 (accessed February 20, 2021).

[80] Rosner R Pfoh G Kotoučova M. Treatment of complicated grief. Eur J Psychotraumatol. (2011) 2:7995. 10.3402/ejpt.v2i0.7995 22893810 PMC3402114

